# Terahertz frequency electronics and photonics: materials and devices

**DOI:** 10.1098/rsta.2023.0378

**Published:** 2025-05-08

**Authors:** Joshua Freeman, Edmund Linfield, Alexander Giles Davies

**Affiliations:** ^1^ School of Electronic and Electrical Engineering, University of Leeds, Leeds LS2 9JT, UK

**Keywords:** quantum cascade laser, two-dimensional materials, transition metal dichalcogenides, topological insulators, semiconductor heterostructures, terahertz electronics and photonics

## Abstract

The terahertz frequency region of the electromagnetic spectrum sits at the interface of electronics and optics, lying between the microwave and infrared (IR) spectral regions. Although there are significant challenges to access, understand and exploit this distinctive region of the spectrum, there are immense benefits in its exploration for both discovery- and challenge-led research, from fundamental studies of laser operation through to the development of new spectroscopy instrumentation. The last 25 years has witnessed remarkable efforts to advance the field of terahertz science and engineering, and this is the subject of this article. Advances in the growth of precisely layered semiconductor materials have enabled a number of new terahertz device technologies, including high-performance quantum cascade lasers (QCLs) and quantum well photodetectors. Recent advances have included the use of thin magnetic films for efficient terahertz generation. We also review the increasing interest in contemporary two-dimensional (2D) materials for terahertz optoelectronic devices. New materials including graphene, topological insulators, transition metal dichalcogenides and novel semi-metals have shown promise as highly efficient terahertz radiation detectors and modulators. Finally, we summarize the challenges which still exist in the field of terahertz electronics and photonics, and how new materials and new device technologies might meet these challenges.

This article is part of the theme issue ‘Science into the next millennium: 25 years on’.

## 1. Introduction

The terahertz frequency region of the electromagnetic spectrum (conventionally 0.3−10 THz ≡ 10−333 cm^−1^ ≡ 1−0.03 mm) lies between the microwave and infrared (IR) spectral regions and, as such, has the intriguing property of sitting at the interface between electronics and optics. Its unique position in the spectrum poses challenges, however, to the development of convenient coherent sources and detectors of terahertz radiation, and hence the ability to study and exploit its properties for fundamental research and for the development of new technologies. In the microwave region, lying to lower frequencies, the power available from electronic devices reduces rapidly at fundamental frequencies substantially above a few hundred gigahertz. This is partly a result of the need for very short carrier transit times, but is also a consequence of the low powers produced by electronic devices with the small active areas necessary to minimize their capacitance. At higher frequencies, the generation of light through the radiative recombination of conduction band electrons with valence band holes across a semiconductor direct bandgap has long been exploited in diode lasers operating at visible and near-IR frequencies. However, Auger recombination of electrons and holes means that this technique cannot be extended to produce mid-IR or terahertz radiation [1].

Notwithstanding, terahertz source technology based on acceleration of electrons such as free-electron lasers, gyrotrons and backward wave oscillators, or through discreet transitions in gases (such as in methanol lasers), have long been available, but their large size, power requirement and experimental flexibility, limits their use to specialist situations. Similarly, detectors of terahertz radiation including thermal bolometers and those based on the pyroelectric effect are long established and are still some of the most effective and sensitive detectors available. For an introduction to terahertz technology, see [2], for example.

The relative inaccessibility of the terahertz region of the spectrum has been known colloquially as the ‘terahertz gap’ for several years. However, this terahertz gap, separating the electronics and optics worlds was recognized a very long time before this. In their 1897 paper ‘Heat rays of great wave length’ [3], for example, Rubens & Nichols wrote, ‘Since we have become accustomed to think of waves of electrical energy and light waves as forming component parts of a common spectrum, the attempt has often been made to extend our knowledge over the wide region which has separated the two phenomena, and to bring them closer together, either by cutting down the wave length of electrical oscillations … or by the discovery and measurement of longer heat waves’. The work of Rubens and others in measuring the temperature dependence of blackbody radiation of ‘great wave lengths’, including at wavelengths in what we would now call the terahertz frequency region, was instrumental in providing Max Planck with the crucial data in 1900 to confirm his hypothesis of what became known as Planck’s radiation law, which heralded the dawn of quantum mechanics [4].

Owing to the difficulties in fabricating compact solid-state sources and detectors of terahertz radiation, researchers focused attention on all-optical techniques, employing near-IR femtosecond pulsed lasers to generate pulses of terahertz radiation through electro-optic rectification in nonlinear crystals or from a device known as a photoconductive switch [5]. The latter comprises a chip of biased semiconductor where the absorption of the femtosecond pulse allows a transient current to flow. A popular material for this is GaAs, which can be carefully engineered and grown below the usual growth temperature to provide the rather counter-intuitive properties of having simultaneously short electron and hole recombination lifetimes, high electron and hole mobilities and a high resistivity [6]. This material, known as low-temperature-grown (LT-) GaAs, was developed in the mid-1980s [7], and the subsequent development of coherent broadband terahertz spectroscopy and imaging systems led to the surge of international activity in this field and arguably started the modern era of terahertz science and technology.

Developments in terahertz technology have also been driven by the wide range of materials that can be explored in the terahertz range, including organic and inorganic crystalline materials and gases, which exhibit sharp characteristic (ro-)vibrational features in the THz frequency range, see [8], for example. The terahertz spectra are also sensitive to both intra- and inter-vibrational molecular modes, making terahertz spectroscopy highly sensitive to small changes in crystalline structure. In many solid-state materials, carrier scattering and dephasing events occur on timescales of tens to hundreds of femtoseconds, making terahertz frequency techniques ideal for studying carrier dynamics, allowing a range of fundamental phenomena to be investigated.

In our previous Perspectives article in 2000 [9], we discussed how commercially driven technological developments in semiconductor materials and electronic devices occurred alongside advances in fundamental physics obtained from investigation of the same semiconductor devices. Perhaps unsurprisingly, a similar narrative (and one that we will explore in this paper) can be told about the modern development of terahertz science and technology. In this article, we will first review the remarkable progress in the growth of sophisticated multi-layered semiconductor materials to create the first compact solid-state lasers operating in the mid-IR [10] and then, in 2002, in the terahertz region of the spectrum [11]. We also discuss detectors, using the same type of heterostructures, but also making use of traditional transistor architectures. We then move on to describe advances in materials for laser-driven terahertz devices, with a focus on recently developed spintronic emitters. In the final section of this article, we switch attention to two-dimensional (2D) and topological materials to examine their applications to, and potential impact on, terahertz science and technology.

## 2. Semiconductor heterostructures

The impact of layered semiconductor heterostructures on science and technology over the last few decades has been enormous. These devices comprise a stack of semiconductor layers, each often just a few nanometres thick, grown on top of each other. The optical and electronic properties of the constituent materials are controllably modified, and quantum mechanical phenomena give rise to new structures with new properties. Our previous Perspectives article highlighted the GaAs–AlGaAs single heterojunction (a heterostructure of just two semiconductor materials, GaAs and AlGaAs), where electrons accumulate at the material interface to form a 2D electron system (2DES). The electrons are trapped in a potential energy well of width similar to their quantum mechanical wavelength with the consequence that the electron energy perpendicular to the interface becomes quantized, as in the textbook ‘particle in a box’ situation. Measurements of these electronic systems can reveal fascinating quantum mechanical phenomena including the Nobel Prize winning integer and fractional quantum Hall effects (QHE) [12,13] (the former first observed in the 2DES formed in silicon metal-oxide semiconductor field-effect transistors (MOSFETs)). Further reduction in dimensionality through lateral pattering of the 2DES to confine the electrons into one-dimensional (1D) channels, rings, dots and other geometries led to the observation of many exciting phenomena and forms the basis of the field of mesoscopic physics [14].

**Figure 1.**
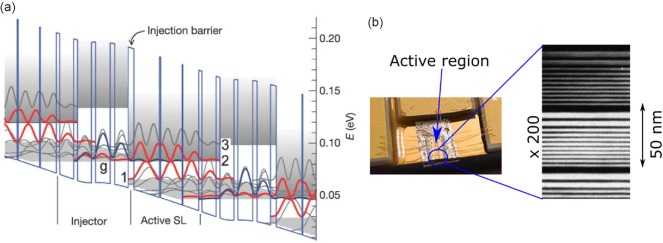
(a) Conduction bandstructure of the first terahertz frequency QCL, presented in reference [11]. The vertical axis shows the electron energy as a function of distance into the device on the horizontal axis. The GaAs layers create a series of potential wells separated by Al_0.15_Ga_0.85_As potential barriers. The widths of the GaAs layers range from 9.9 to 18.8 nm, and it is a consequence of the quantum confinement caused by the narrow wells that the electron energy is split into a series of minibands that are represented in the figure by their corresponding wavefunctions (moduli squared). The bandstructure is shown under electrical bias, and electrons traverse through the minibands from left to right with the terahertz lasing transition occurring between the red states, labelled 2 and 1. The figure shows just two periods of the overall device; 104 periods were grown in the original study. (b) Photograph of a QCL device, here the active area is 250 µm x 3 mm, together with a transmission electron microscopy image of the GaAs/AlGaAs layers that form the wells and barriers in the active region.

Heterostructured semiconductors also find an important role in optoelectronic devices. They can greatly improve the efficiency of diode lasers and LEDs by confining carriers to the active regions, and reduce unwanted photon reabsorption, enabling their widespread adoption. Here, the charge carriers are driven ‘vertically’ through the semiconductor layer stack, rather than primarily within the layer as is the case in transistors or in the study of the quantum Hall effect, for example. In the visible and near-IR spectral regions, direct bandgap semiconductor diode lasers make excellent emitters. However, as the wavelength increases and the required semiconductor bandgap decreases, the non-radiative Auger recombination between electrons and holes reduces the efficiency [1], and alternative emission (and detection) schemes must be found. A solution is to move from interband transitions in bipolar devices, where conduction band electrons recombine with valence band holes to emit photons, to intersubband transitions in unipolar devices, where electrons transition between energy levels entirely within the conduction band. The first intersubband laser, known as the quantum cascade laser (QCL), was demonstrated in 1994 by Faist *et al*. [10]. Emitting in the mid-IR, around 4 µm, it was based on a multilayered Ga_0.47_In_0.53_As–Al_0.48_In_0.52_As heterostructure, lattice matched to InP. The electron subbands result from the particle-in-the-box-type quantum confinement created by the nanometre-thick semiconductor layers, with the subband separation, and hence the wavelength of the emitted photons, tuned by the heterostructure design. In 2002, Köhler *et al*. reported [11] the operation of a QCL emitting at 68 µm in the terahertz region, composed of GaAs wells and Al_0.15_Ga_0.85_As barriers on a GaAs substrate; the active-region heterostructure is shown in figure 1.

### (a) Quantum cascade lasers

Crucial to the development of semiconductor heterostructures has been the advancement of sophisticated semiconductor materials growth technologies, with molecular beam epitaxy (MBE) perhaps the best known. MBE is a form of high vacuum evaporation, where pure elements evaporated in ultra-high vacuum impinge on a substrate, allowing the fabrication of near-perfect crystals with the crystal lattice maintained throughout (known as epitaxial growth), and enabling extremely abrupt changes in composition and doping. By careful selection of the substrate to minimize strain, high-quality multi-layer crystalline films can be grown epitaxially. While there are alternative epitaxial techniques such as metalorganic chemical vapour deposition (MOCVD) that are more economical and can be used for many structures such as diode lasers, quantum dots and mid-IR QCLs, MBE is still the preferred choice for terahertz devices such as QCLs and quantum well IR photodetectors (QWIPs), owing to the unrivaled control over layer thicknesses and doping levels [15].

The choice of materials for the wells and barriers of the heterostructure is also very important. For a stable crystal the lattice constant of the two materials must match closely; the closer they match, the thicker the crystal that can be grown. For terahertz QCLs, where thick heterostructures are desirable to increase the laser gain, the AlGaAs/GaAs material system is preferred because of the near-perfect lattice match between these materials that results in very high-quality crystals with few lattice defects. This enables very thick structures with 1000s of monolayers to be grown, with total thicknesses to 24 µm [16]. Furthermore, there is a clear link between the level of background doping and impurities in the growth chambers and the resulting laser performance [17,18].

The maturity of the crystal growth and the demonstration of the QCL scheme at terahertz frequencies gave researchers a high degree of flexibility to choose the thicknesses and alloy compositions of the wells and barriers in the heterostructure active region in the search for improved designs and performance. The first design, shown in figure 1 [11], was based on a chirped superlattice, where the electron states form ‘minibands’ and the laser transition is from the bottom of one miniband to the top of the next, downstream miniband. However, this only lased up to a maximum temperature of 50 K. Since then, there has been an intense research effort across a number of research groups to improve this operating temperature. ‘Bound-to-continuum’ designs soon followed in 2003, where the upper miniband of electronic states in the chirped superlattice design is replaced by a single ‘bound’ state, but the lower miniband is retained, immediately below the lower laser level, providing a larger number of states for the electrons to scatter into. The first realization of this design, by Scalari *et al.*, increased the operating temperature to 90 K [19]. An alternative scheme, proposed by researchers from MIT [20], and known as the ‘resonant phonon’ design, aimed to use the efficient scattering when the separation between electron states is resonant with the longitudinal optical phonon energy (36 meV in GaAs) to depopulate the lower lasing level and improve the population inversion. Because the phonon population increases with temperature, these designs are more robust against increasing temperature, immediately improving the operating temperature to 137 K. Over the last decade refinements to these designs have resulted in the maximum operating temperature of terahertz QCLs increasing to 261 K [21], within the range of compact solid-state Peltier coolers. A drawback of this type of design, however, is the large driving currents typically needed, requiring the QCL to be electrically pulsed and hence leading to a pulsed emission. For many applications, ‘continuous wave’ mode (DC-powered) is preferred. Here, hybrid designs, making use of both minibands and resonant phonon extraction [22,23], offer a good compromise, allowing efficient continuous wave operation at temperatures within the range of compact cryocoolers. The emission frequencies of terahertz QCLs currently span 1.3–5.4 THz [24] with peak output powers reaching 2.4 W in pulsed operation [16]. In addition to controlling the thickness of the well and barrier layers in QCL heterostructures, MBE growth also enables controlled placement of dopant atoms, which is important for laser performance [25].

Clearly, the operating temperature of terahertz QCLs is a barrier to widespread application, and while the low photon energy makes operation close to room temperature challenging, operation is not restricted to a fundamental limit such as Boltzmann’s k_B_T owing to the non-equilibrium laser dynamics.

While the GaAs/AlGaAs materials system remains by far the most common choice for QCLs in the terahertz region, and has achieved the highest performance to date, other material systems may provide potential advantages such as lower effective electron masses and higher phonon energies. These could lead to higher operating temperatures. This has stimulated research into other material systems, with QCLs realized in InGaAs/InAlAs/InP [26] and InGaAs/GaAsSb/InP [27], while there has also been electroluminescence observed from GaN/AlGaN [28] and ZnO/ZnMgO [29] devices. However, to-date none has surpassed the performance of the AlGaAs/GaAs material system [24].

Semiconductor heterostructures have also been used to improve the speed of electronic devices, enabling them to extend their emission frequency up into the terahertz range. A device closely related to, but pre-dating, the QCL is the resonant tunnelling diode [30]. These devices also make use of vertical transport in a semiconductor superlattice, relying on resonant tunnelling between confined electron states, as their name suggests. This strong resonance appears in the I-V characteristic of the device as a region of negative differential resistance. By embedding the device in an electronic resonator and biasing the device in the negative differential resistance region, oscillators with frequencies from 0.1 to 2 THz can be realized. While the power in the 1−2 THz range is typically no more than a few µW, room-temperature operation and low operating power requirements make these devices attractive for applications such as wireless communications. There has also been improvement in the operation frequency of silicon-based semiconductor devices in recent years [31]. Improvements in fabrication techniques have allowed a reduction in device dimensions in well-established material technologies such as complementary metal-oxide semiconductor (CMOS), bipolar CMOS and SiGe heterojunction bipolar transistors. These reduced dimensions, driven by the search for increased efficiency in computer chips, have enabled increased bandwidths to be extracted from these materials, for example, 0.7 THz emitters fabricated from a 65 nm CMOS process [32]. Nevertheless, it seems unlikely that silicon-based materials will prove to be efficient emitters above 1 THz.

### (b) Intersubband detectors

**Figure 2.**
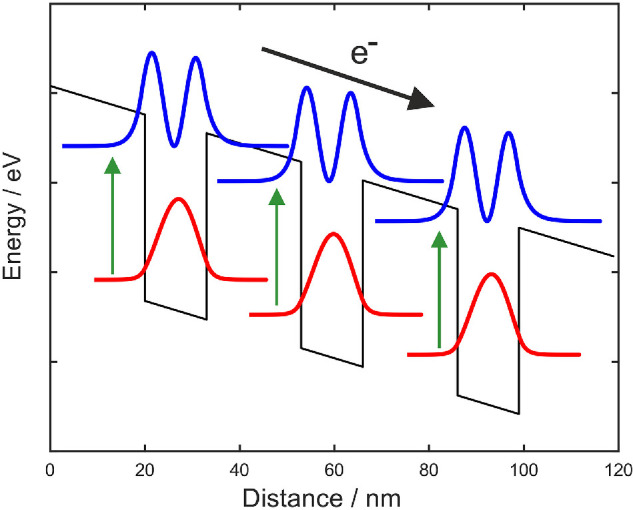
Schematic diagram of a QWIP bandstructure. Each quantum well has one bound state in the well (wavefunction moduli squared in red) and a quasi-bound state close to the electron continuum (blue). Electrons in the ground (bound) state move to the quasi-bound state after absorbing photons with the corresponding energy (green vertical arrows). These then tunnel or scatter into the continuum and can be detected as a photocurrent, black arrow.

**Figure 3.**
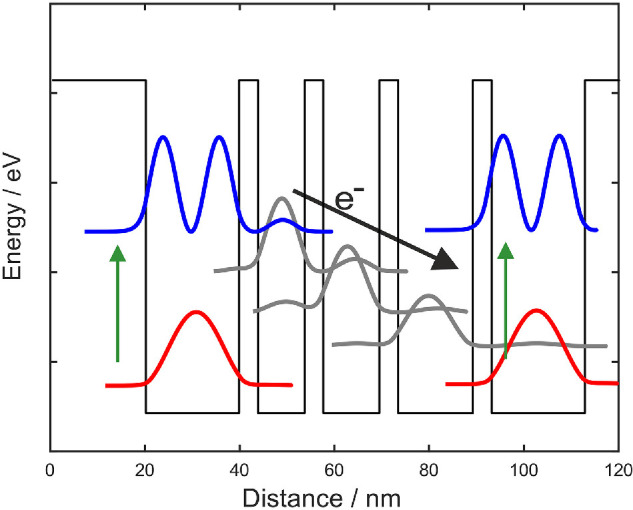
Quantum cascade detector. Each period has four wells and corresponding barriers. The photon is absorbed (green vertical arrow) between the ground (red) state and excited (blue) state. Once in the excited state, electrons can tunnel or scatter via intermediate states (grey) to the next period, contributing to the photocurrent.

Intersubband transitions in semiconductor heterostructures can also be used to detect long wavelength photons. These detectors are typically known as QWIPs, and have been studied since the 1980s for mid-IR detection [33,34], pre-dating QCLs. This detection scheme has also been extended to the terahertz region, where GaAs/AlGaAs heterostructures are the most commonly used. However, to operate on the same principle as mid-IR QWIPs, where the first excited state is close to the top of the barrier (figure 2), a very low aluminum mole fraction is required in the Al_*x*_Ga_(1 − *x*)_As, around *x* = 0.01−0.05 compared to around *x* = 0.15 for QCLs; these low barriers mean operation is restricted to very low temperatures [35,36]. More sophisticated active-region designs that present the excited electrons with a number of bound states to scatter into as they progress to the next period, known as quantum cascade detectors (figure 3) [37], have also been developed and reduce the dark current compared to single well QWIPs.

Beyond GaAs/AlGaAs, other materials have been explored, for example, the GaAs/InGaAs material system used in the mid-IR has shown detection down to 15 µm [38], but the barrier height makes extension to terahertz frequencies challenging. Quantum cascade detectors have been realized in GaN/AlGaN at 5.7 THz [39]; however, the efficiency and operating temperatures achieved have not improved significantly on GaAs/AlGaAs devices

An alternative approach to improve performance is to increase the confinement of the carriers, moving from quantum wells to 1D channels (nanowires) and ultimately to full three-dimensional (3D) confinement (quantum dots). Rather than the lateral etching or electrostatic patterning that is used to achieve this in the 2DES mesoscopic systems mentioned earlier, here, advanced growth techniques are typically used to realize a ‘wire’ or ‘dot’ of one semiconductor, encapsulated within another. The reduction in the carrier degree of freedom increases its lifetime [40], which should lead to higher efficiency detectors. While quantum dots have been studied for a number of years in the near- and mid-IR, the extension to intersubband transitions in the terahertz region requires challenging growth because of the larger dot dimensions required. Nevertheless, operation of terahertz quantum dot detectors has been reported [41] and shows higher operating temperatures (up to 150 K), but controllable growth remains a challenge. There are other quantum confined detectors, including ‘quantum rods’ [42] and ‘quantum ratchet’ [43] designs, that all make use of semiconductor heterostructures.

An advantage of the intersubband tunnelling and scattering in QWIPs and related intersubband detectors, is the rapid electronic transport enabled, leading to high-speed operation, with up to 6.2 GHz at 4.2 THz reported [44]. However, the intrinsic speed is expected to be in excess of 20 GHz. In the mid-IR, this has led to the demonstration of all intersubband communications systems, comprising a QCL, intersubband modulators and QWIPs [45]. While the same principles could be applied to realize a terahertz frequency communications system, the low temperatures currently required for the modulators and QWIPs would be prohibitive for most applications.

Competing technologies for high-speed terahertz detection include hot-electron bolometers (HEBs), capable of detection bandwidths of a few GHz in the terahertz range. For example, superconducting NbN HEBs have been demonstrated at 4.7 THz [46]. They operate in a similar way to conventional pn-junction diodes with a similar rectifying electrical characteristic, but operate at lower forward voltages and have very fast transit times. However, the requirement for superconducting behaviour means that their operating temperature is fundamentally limited. An alternate high-speed detector, but operating at room temperature, is the Schottky diode. These are typically fabricated from GaAs, owning to the very high electron mobility and high-quality growth, and provide detection to over 5 THz, with bandwidths greater than 10 GHz, at room temperature. High noise levels, however, require the use of powerful sources and local oscillators [47,48]. While terahertz Schottky diodes are commercially available [49,50], their use is mostly restricted to frequencies below 2 THz as the noise levels increase (and the conversion efficiency decreases) quickly above this, and fabrication becomes very challenging.

Beyond the choice of the material systems, recent advances in QWIPs have been made by combining these detectors with subwavelength engineering, inspired by metamaterial concepts. Metamaterials typically exploit the subwavelength patterning and structuring of materials and devices to change the properties of the bulk material. This technique is well suited to the relatively large-wavelength terahertz region, where subwavelength engineering at the micrometre is well developed. Metamaterial devices also provide the benefit of typically being very thin, reducing otherwise bulky optics. When coupled with QWIPs, these techniques have been used to increase the effective detector absorption area while reducing the electrical area to minimize dark current [51–53], allowing operation up to 60 K, compared to approximately 20 K for traditional geometries. These principles can also be applied to terahertz modulators to control and concentrate radiation into active areas, thereby increasing efficiency [54].

### (c) FET-based detectors

Interactions of terahertz photons with confined electrons is not the only way to detect radiation. There is a long history of sensing terahertz radiation by using the temperature change it induces in a material. Many physical effects have been harnessed to measure this change, from the expansion of a gas (Golay cells), change in electrical resistance (bolometers), or more direct methods such as using the pyroelectric effect or via thermopiles [55]. While some of these detectors offer the highest sensitivity commercially available (most notably, cryogenically cooled bolometers), all are intrinsically ‘slow’ due to the thermal detection mechanism.

More recently, the search for sensitive room terahertz detectors has explored the interaction of terahertz radiation with the collective motion of electrons, known as plasmons, with particular focus on using the 2DES in semiconductor field-effect transistors (FETs), the same type of device that was central to the development of mesoscopic electronics and the discovery of the integer and fractional QHE decades earlier. The detection mechanism was first proposed theoretically by Dyakonov & Shur [56], with the first experimental demonstrations reported in 2002 [57,58]. The 2DES in the source-drain channel of the FET supports plasma waves that are modified by the incoming terahertz radiation, typically coupled to the channel by an antenna-shaped gate electrode. These so-called ‘TeraFETs’ have now been fabricated from a number of semiconductors including GaAs/AlGaAs [59], InAlGa/InGaAs [60], AlGaN/GaN [61–65] and silicon CMOS [66–70]. More sophisticated theoretical models have also been developed that take the change of the impedance of the channel by plasmonic effects into account [71].

Some of the most advanced FET detectors are those based on silicon CMOS, typically fabricated in commercial semiconductor foundries. Here, the research is accelerated by the existing technology platform, benefiting from a sophisticated process technology, integrated read-out circuitry and the potential to fabricate detector arrays for imaging [72,73]. State-of-the-art detectors show noise-equivalent powers (a measure of detector sensitivity) of 20 pW Hz^–1/2^ below 1 THz [74] and can detect frequencies up to 30 THz [75]. While the response times reported to date have been relatively long, corresponding to bandwidths in the kHz range, this is limited by the readout electronics, and the devices should be intrinsically fast enough to reach sub-nanosecond response times.

## 3. Materials for laser-driven terahertz frequency generation

**Figure 4.**
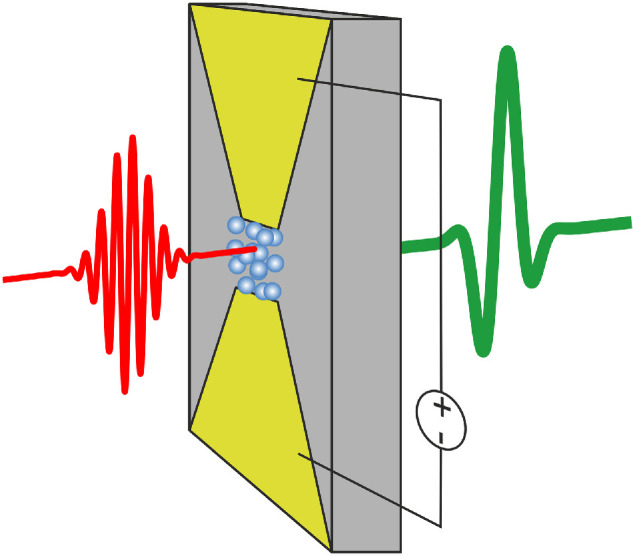
Schematic diagram showing operation of a photoconductive emitter. A femtosecond pulse (red, left) is focused on to a semiconductor surface, generating electron-hole pairs. The applied bias accelerates the charges (shown as spheres, blue), resulting in a terahertz pulse (green, right).

As mentioned in §1, the use of near-IR femtosecond pulsed lasers to generate broadband terahertz pulses is an enabling technology and the most widely used system for spectroscopy studies in this region of the electromagnetic spectrum. There has been continuing work on photoconductive terahertz sources (figure 4) over the last three decades with research focusing on a number of areas, including the search for new semiconductor materials with improved electrical characteristics. LT-GaAs is still widely used because of its short carrier lifetimes and a bandgap matched with the 800 nm pulses produced by ultrafast titanium-sapphire lasers. However, the increasing availability, reduced cost, convenience and high power of Yb- and Er-doped fibre lasers operating at approximately 1 and 1.55 µm wavelength, respectively, has motivated intensive research into materials with compatible bandgaps and suitable electrical characteristics. InGaAs has a well-matched bandgap of 1.55 µm, but suffers from low dark resistance, although this can be overcome by doping compensation [76] or through use of heterostructured devices [77]. Another key driver for the development of photoconductive emitters is the desire to generate high-field terahertz pulses for studies of nonlinear spectroscopy exploiting high pulse energy amplified laser systems, which have traditionally used nonlinear optical crystals as the emitter. The active area of the photoconductive devices can be increased to avoid saturation by using interdigitated arrays of biasing electrodes, while keeping the applied biases relatively modest. These designs also offer the ability to control polarization of the generated THz pulse by structuring the electrodes [78].

**Figure 5.**
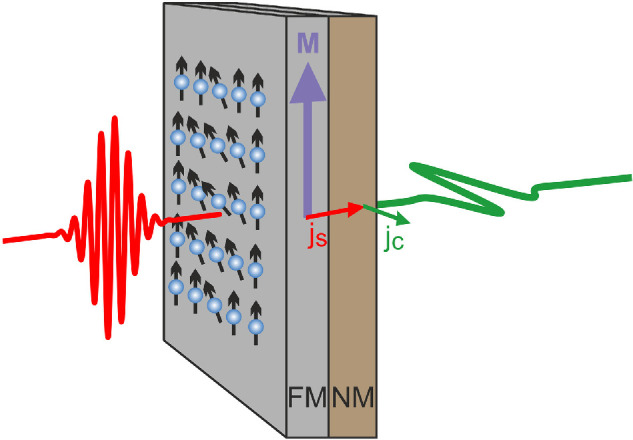
Diagram of spintronic terahertz emitter, from reference [79]. A femtosecond pulse (red, left) is focused on to a FM film with aligned spins. A spin current (*j*_s_) diffuses into the NM layer, generating a terahertz pulse (green, right).

More recently, a new pulsed laser-driven terahertz source has emerged. In a similar way to the photoconductive emitters described above, a terahertz pulse is generated from a transient current; however, the mechanism to generate this current is very different. Typically known as ‘spintronic emitters’, the devices are composed of two or more thin metallic layers, a ferromagnetic (FM) layer and a non-magnetic (NM) layer (figure 5). Although this effect was first observed in 2004 by Beaurepaire *et al.* [80], it was work by Kampfrath *et al.* in 2013 [81] and others [82] that showed that these devices are viable emitters. The emission is initiated by a femtosecond laser pulse, visible or near-IR, that is directed on to the sample and creates a non-equilibrium population of spin-up and spin-down carriers in the FM layer. These diffuse, causing a spin-polarized current to enter the NM layer, which is converted to a charge current in a process dominated by the inverse spin Hall effect. The resulting charge current is perpendicular to both the spin current and the coercive magnetic field. Typical devices have layers that are just a few nanometres thick, which coupled with the ultrafast spin dynamics, leads to terahertz pulses with very large bandwidths, over 30 THz [83], and ideal for spectroscopy applications. Spintronic emitters have been realised in a wide range of FM and NM materials, with the field and bandwidth of the terahertz emission dependent on the choice of the two materials. For the FM layer, Fe, Co, Ni and related alloys all show emission, with some of the most efficient generation from the CoFeB alloy. For example, Sasaki *et al.* [84] found that for Ta/(Co_*x*_Fe_1 − *x*_)_80_B_20_ layers, *x* = 0.1−0.3 was the optimum for terahertz generation, and also showed a maximum in the saturation magnetization. The NM material has also been investigated; here a large spin–orbit interaction appears to be beneficial, with terahertz emission correlated with the spin Hall conductivity. Most work has found Pt to be the most efficient NM material. Currently, the most efficient spintronic emitters make use of a tri-layer material structure: W/Co_20_Fe_60_B_20_/Pt with each layer only approximately 2 nm thick. With this layer structure, together with a thermally conductive silicon substrate and large area generation, peak fields of up to 1.5 MV cm^−1^ have been reported [85].

## 4. 2D materials

The discovery of graphene in 2004 [86] generated a surge of interest in this and other 2D materials. These materials, so called because they are only a single atom or a few atoms thick, confine electrons to a 2D environment, not dissimilar to the 2DESs formed in the GaAs–AlGaAs single heterojunction or the silicon MOSFET discussed above. However, in graphene the bandstructure is quite different, distinct from the well-known bandstructures present in metals and conventional semiconductors. This results in a range of exotic electrical, optical and mechanical properties, which enabled, for example, observation of the quantum Hall effect at room temperature by Novoselov *et al.* in 2007 [87]. Here, we focus on the most important 2D materials and their applications to terahertz technology and refer the reader to dedicated reviews for further details [88–90].

### (a) Graphene

**Figure 6.**
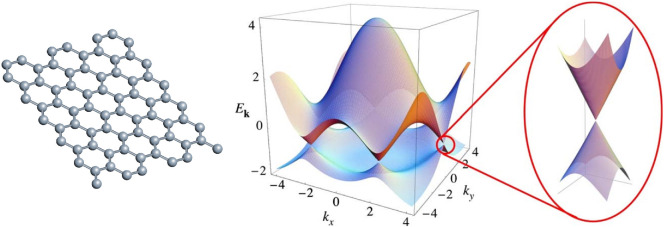
(Left) The hexagonal crystal structure and (right) electronic dispersion in a single layer of graphene showing the six gapless ‘K’ points. The magnified region shows the linear dispersion, characteristic of massless carriers that lies close to the K points. Figure reproduced from [91].

Graphene, the most widely studied 2D material, has a simple crystal structure consisting of carbon atoms arranged on a 2D hexagonal lattice. This results in an electronic bandstructure of a zero-gap semiconductor, but with linear dispersion near the ‘Dirac point’ where the bands meet, resulting in massless electrons dominating the electrical properties (figure 6). Perhaps more important for terahertz applications are the ability to control the Fermi level by an electrostatic gate, the rapid carrier dynamics, and an exceptionally high absorption dominated by free-carrier absorption in the terahertz range and independent of frequency (2.3% per monolayer) at higher frequencies. The frequency at which this transition occurs is dictated by the carrier density [92]. These features make graphene, and other related 2D materials, well suited to applications in terahertz detectors and modulators [93,94].

When used as a detector, there are three main effects to consider in graphene: the photo-bolometric effect, the photo-thermoelectric effect, and plasma wave rectification. The high terahertz absorption, exceptionally low specific heat, and ultra-fast carrier thermodynamics lead to the generation of large thermal gradients and make graphene well suited to bolometric detection. The only drawback is the relatively slow variation in conductivity with temperature. One successful strategy to overcome this, demonstrated by Lee *et al.* [95], was to integrate Josephson junctions and measure the low-temperature switching currents. While this particular type of graphene bolometer is intrinsically limited to superconducting temperatures, the ultra-low noise levels (30 zW Hz^-1/2^) [96] mean these detectors could find applications in tests of quantum electrodynamics or in quantum computers for qubit readout [97]. An alternative approach, explored by Duan *et al*., is to make use of the large Seebeck effect that is present in high-quality graphene [98] to convert a thermal gradient directly into a voltage via the photo-thermoelectric effect. Here, to sustain the thermal gradient, asymmetric contacts, antenna structures or gratings can be introduced [99].

**Figure 7.**
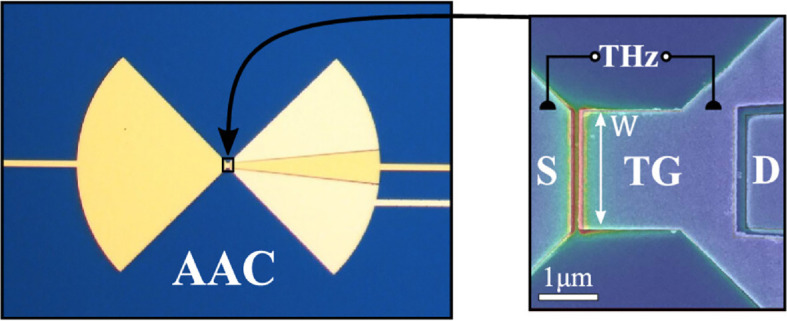
Image of an asymmetric antenna-coupled G-FET detector, including (right) a magnified image of the central region. ‘S’ denotes source, ‘D’ denotes drain and ‘TG’ denotes top gate. The colour-shaded overlay on the scanning electron microscopy image represents the simulated terahertz electric field distribution at 400 GHz calculated with the Maxwell solver CST Studio Suite. The single-layer graphene sheet (not visible) sits below the electrodes. Reprinted with permission from [104]. Copyright 2024 American Chemical Society.

Graphene has also been widely used in FET geometries for detectors [100–103] (figure 7). While these detectors appear to operate in a similar way to the conventional semiconductor FETs described above, recent work by Ludwig *et al*. [104] has shown that the terahertz response has two components: a resistive self-mixing from carrier density oscillations and a second component caused by carrier heating and the photothermoelectric effect. At higher frequencies, greater than 2 THz, the latter is the dominant rectification mechanism.

Besides applications as a detector, the properties of graphene are also well suited to use in electrically controlled modulators, making use of a conductivity that is widely tunable by gate bias. While modulation can be achieved using relatively simple geometries [105], the modulation speed tends to be limited by the capacitance of the large active area. These limitations can be overcome by carefully engineering the device area and incorporating resonators to enhance the effects, resulting in modulation speeds greater than 3 GHz [106].

The unusual carrier thermodynamics in graphene also gives rise to some extreme nonlinear effects. These include saturable absorption where the absorption of a material reduces with increasing power density. Most materials that exhibit this only do so in a narrow frequency range. However, in graphene, this phenomenon is observed over a very wide range, extending from the optical [107] to the terahertz region [108]. High harmonic generation is another application of graphene that is developing in the terahertz frequency range, first reported by Hafez *et al.* [109] (see also discussion on harmonic generation in topological insulators below). It can be explained by a thermodynamic model [110], relying on the fast electron momentum scattering time in graphene which is on the order of 10−100 fs, one order-of-magnitude faster than the terahertz wave period. More recently, the same effect has been used to observe third harmonic generation from 3.2 to 9.6 THz using a THz QCL pump source [111].

### (b) Transition metal dichalcogenides

Looking beyond graphene, there is a range of other 2D materials which have generated a lot of interest in recent years, most notably transition metal dichalcogenides (TMDs) [112]. These are 2D layered semiconductors with the chemical formula MX_2_, where M is a transition metal atom (e.g. Mo, Pt or W) and X is a chalcogen (e.g. S, Se or Te). Their electronic properties are widely tunable by varying the constituent elements or the number of layers; MoS_2_ is the most widely studied of these materials, but its application in the terahertz region is more limited because of its large bandgap and lower mobility (compared to graphene).

**Figure 8.**
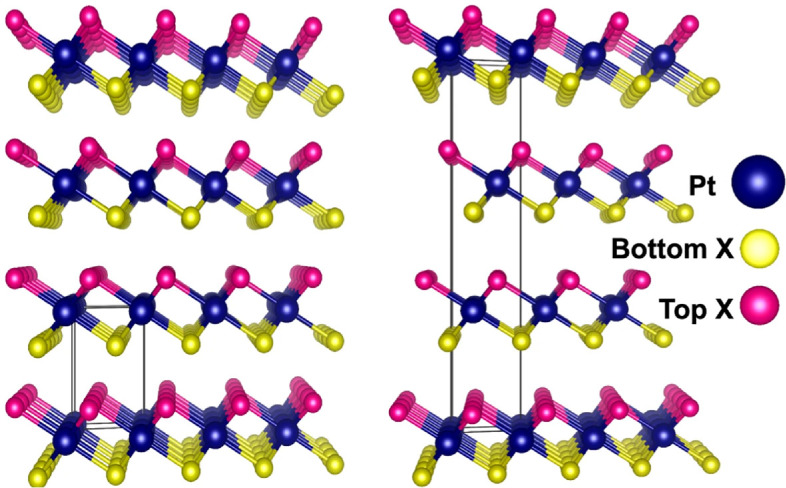
Layers of the transition metal dichalcogenide PtX_2_, where X is S, Se or Te. The number of layers in the stack controls the size of the bandgap. Reprinted from [113] under Creative Commons CC BY license.

**Figure 9.**
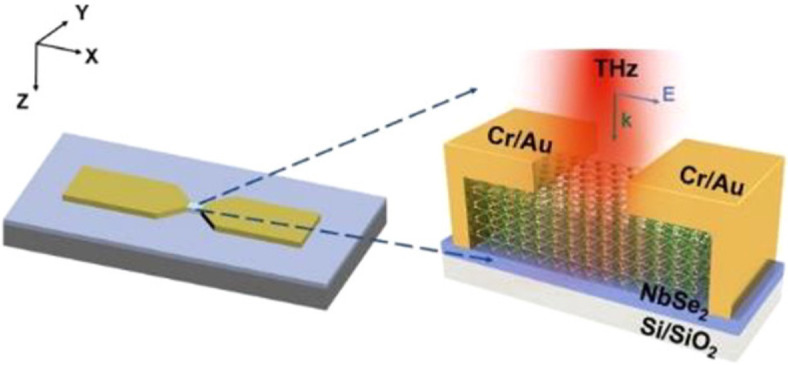
Terahertz detector based on NbSe_2_. The terahertz radiation is coupled via an antenna into the NbSe_2_ material, where it is detected via the photoelectric effect. Reprinted with permission from [119]. Copyright 2022 American Chemical Society.

A TMD that has received more interest for terahertz applications is PtSe_2_ because of its higher carrier mobility, adjustable band gap of 0−1.2 eV and fast response times [113,114] (figure 8). This has been used to realize high-efficiency terahertz modulators in PtSe_2_ films [115]. Terahertz photodetectors have also been realized in simple PtTe_2_ planar geometries for direct detection of terahertz photons, resulting in high responsivities of 1.6 AW^−1^ and noise-equivalent powers less than 10 pW Hz^−1/2^ [116]. The related material, PdSe_2_, has also been used to make FET detectors for the terahertz frequency range [117], while NbSe_2_ has been integrated into HEB detectors [118] and used for terahertz photoelectric detection at room temperature, with a quoted noise-equivalent power reaching 5.4 fW Hz^–1/2^ at 0.173 THz [119] (figure 9). However, care must be taken when comparing responsivities and noise-equivalent powers in the literature; for example, [116] uses the device area (4800 µm^2^), whereas [119] uses only the area of the active material (32 µm^2^) when calculating these values.

The remarkable properties of these materials, combined with an explosion of research driving improvements in material quality, has also led to exploration of how these materials can improve already established devices. For example, combining TMDs with optically driven silicon modulators greatly increases the terahertz modulation efficiency [120], and by replacing the NM layer (e.g. Pt) in spintronic emitters with PtTe_2_ increases the emission efficiency by making use of the larger spin Hall conductivity in this TMD [121].

### (c) Black phosphorus

Another 2D layered material, which exhibits properties intermediate between graphene and TMDs, is black phosphorus (bP). Similar to graphene, its structure is a 2D honeycomb, with van der Waals bonds between layers, but unlike graphene, the layers are ‘puckered’ or corrugated, inducing a large anisotropy [122]. It has a thickness-tunable direct band gap, ranging from 0.3 eV in bulk material to 2 eV for a single layer [123], which is intermediate to the zero bandgap of graphene and the large bandgaps of most TMDs. It also exhibits high room-temperature mobility, greater than 1000  cm^2^ V^-1^ s^-1^ for bulk material, much higher than most TMDs, although not approaching that of graphene. These properties have driven interest in bP for terahertz detectors, with reports of direct detection [124] and exploiting the FET geometry [125]. Gate-tunable anisotropic plasmon polaritons have been measured by terahertz microscopy [126], highlighting the potential of bP for sensing and quantum technology. Despite this potential, serious challenges remain in the fabrication and stability of bP. Oxidation of bP causes a rapid deterioration in its dielectric properties and for this reason, hexagonal boron nitride is regularly used to encapsulate bP. The wafer-scale fabrication of bP lags behind graphene and some TMDs, such as MoS_2_, despite recent progress in pulsed laser disposition of bP [127] and in other growth techniques [128].

### (d) Topological insulators

**Figure 10.**
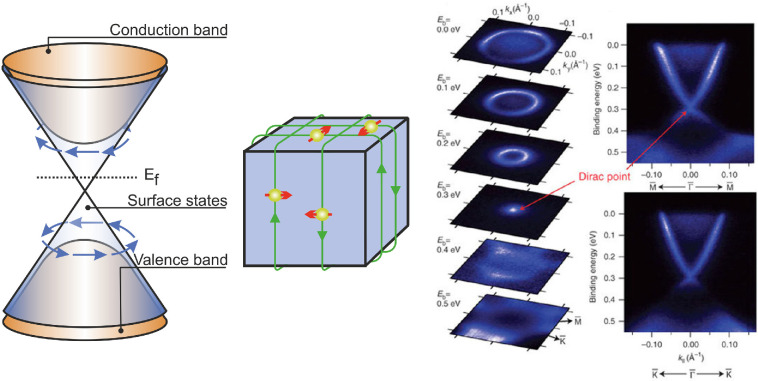
(Left) Schematic diagram showing the bandstructure of a topological insulator. The conduction and valence bands are associated with the bulk of the material, while the cone, with linear dispersion, describes the surface states. The arrows indicate the carrier spin. (Middle) A real space representation of a topological insulator, showing spin–momentum locked surface states, inspired by [133]. (Right) Angle-resolved photoemission spectroscopy measurements of a BiSe_2_ surface after cleaving. These measurements reveal the bandstructure, including the Dirac cone and Dirac point, taken from [134].

Much of the physics of topological insulators (TIs) has origins in the study of the integer quantum Hall effect (QHE) [12], discussed in our previous Perspectives article [9]; the QHE in a 2DES can be interpreted using the concept of topology using TKNN theory [129,130]. More recently, in 2005, 3D topological insulators were proposed by Kane & Mele [131,132], which are characterized by an insulating bulk and conductive topological surface states (TSSs), which follow a linear dispersion and behave as 2D Weyl (also known as ‘Dirac’) fermions (figure 10). This special surface state arises from the very large spin–orbit interaction in these solids that inverts the bulk bandstructure, moving the valence-band-like states above the conduction band. These then ‘unwind’ at the interface with any conventional insulator. The TSSs exhibit spin–momentum locking, where the direction of spin is locked to their momentum, which is not unlike the chiral edge-state conduction that underpins the robustness of the QHE in 2DESs. This gives protection against dissipative back-scattering, leading to the observed TSS high mobilities [135]. The most widely investigated materials are Bi_2_Se_3_, Bi_2_Te_3_ and alloys in the (BiSb)_2_(SeTe)_3_ material system.

As is the case for many new materials, terahertz spectroscopy was initially used to study the material, with reports of a large Kerr effect [136], a polarization dependent photo-galvanic effect [137], and topological magnetoelectric effects [138], as well as being used to study the crystal structure and prominent phonons [139]. There have also been reports of these materials being used as detectors of terahertz radiation [140], but the performance is typically not as high as graphene or some of the other 2D materials mentioned above.

The generation of terahertz radiation in these materials by an incident pulsed near-IR laser has also been investigated, and although the electric fields generated are too small to compete with photoconductive or spintronic emitters, the sensitivity of the generated terahertz to the polarization of the optical excitation and to the crystal orientation has provided an additional tool to study TI materials. Early works, e.g. by Hamh *et al.* attributed the observed circular anisotropy in the terahertz radiation distribution in Bi_2_Se_3_ to the circular photon drag effect [141]. Other mechanisms have been discussed [142,143], and the variation with chemical composition investigated [144]. More recently, Fang *et al.* [145] found that nonlinear surface currents dominate the terahertz emission from Bi_2_Te_3_ under pulsed laser excitation.

**Figure 11.**
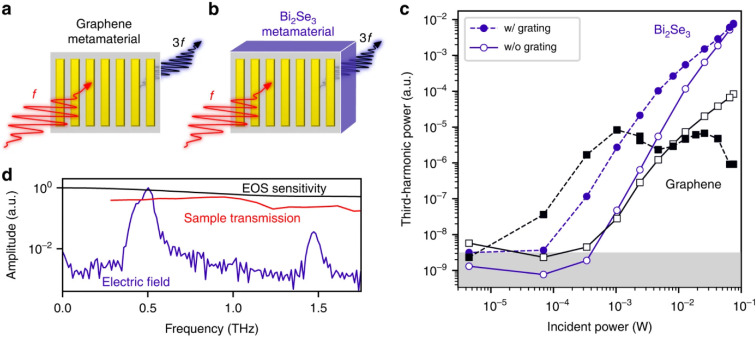
Harmonic generation on the surface of the topological insulator Bi_2_Se_3_. (a,b) Diagrams of the experimental geometry indicating the incident fundamental frequency and the generated 3rd harmonic. (c) The power generated from both graphene and Bi_2_Se_3_ materials. (d) The spectrum of the measured 3rd harmonic signals. Reprinted from [146] under CC-BY licence.

Perhaps more relevant for use in applications of terahertz radiation, harmonic generation has also been reported from the surfaces of TIs (figure 11). The first report, in 2012 [137], was of second harmonic generation (SHG), although the theoretical mechanism for this was initially not clear. More recent work on SHG at optical frequencies indicates the need for symmetry breaking to observe SHG in this material [147]. Third harmonic generation in Bi_2_Se_3_ was experimentally studied [148] and found to exhibit estimated efficiencies as high as 1% for an incident field of 300 kV cm^−1^ [149] and powers into the mW range [146] (figure 11). This efficient generation is thought to be mediated by ultrafast Dirac fermion relaxation in a similar mechanism to graphene [149].

A key motivation for the study of topological insulators is the spin–momentum locking, leading to a spin-textured surface, and proposals for applications in quantum information and spintronics [150]. Given the already strong interest from researchers [151], it is likely that extra degrees of freedom in spin and polarization will also be harnessed for terahertz technology. Combining TIs with the techniques and devices developed for spintronic emitters appears fruitful [152].

### (e) Topological semi-metals

More generally, materials such as graphene, some TMDs and TIs can be classified as topological semi-metals. The key feature of these materials is a gapless crossing in the electronic bands [153] and can appear in either 3D [154] or 2D [155] materials. These materials offer great potential for applications in the terahertz region owing to a strong light–matter interaction and often high carrier mobility, making them ideal for detectors and modulators [156,157], as described above. However, less well-known members of this family have also been investigated, such as the 3D Dirac semimetal Cd_3_As_2_, which has been used as terahertz photodetector [158] and as an optical modulator [159]. Weyl semimetals, such as NbIrTe_4_, also show promise as terahertz detectors, using, for example, the photo-thermo electric effect [160].

Currently, with the theoretical understanding and material growth and quality still under active development, there appears to be considerable potential for these materials to play a role as detectors and modulators in the terahertz region in the future.

## 5. Conclusions and perspectives

In this article, we have described some of the most important developments in materials for terahertz science and technology over the past two decades. In this time, access to terahertz frequency spectroscopy has greatly increased, driven mainly by the availability of pulsed laser-driven time-domain spectroscopy using relatively inexpensive semiconductor-based photoconductive emitters and detectors. These spectroscopy systems have also found industrial applications in process and quality control, in the automotive and pharmaceutical sectors, for example [161]. The specialized sources and detectors for these systems are also still improving, by controlling lifetimes and resistivities in GaAs and InGaAs initially, and more recently through the invention of spintronic emitters.

There has also been great progress on compact sources and detectors of terahertz radiation. While there has been progress on electronic and laser-driven sources in the 0.5−2 THz range, the realization of QCLs has provided powerful narrowband terahertz lasers, covering the 2−5 THz range for the first time. The detection of terahertz radiation has benefitted from the same type of semiconductor heterostructures, namely QWIPs, which are capable of very fast rise times, albeit at very low temperatures. Since then, FET-based detectors have been developed in conventional semiconductors, such as silicon, which benefit from mature fabrication technology. Key advantages of these detectors include their room-temperature operation and low noise. More recently, graphene, some TMDs and bP have shown great promise for room-temperature terahertz detectors and modulators.

For the 2D materials described here, fabrication maturity is still a long way from that of conventional semiconductors, and especially for terahertz applications which require large material areas. While this is improving rapidly, particularly for graphene and TMDs such as MoS_2_ where chemical vapour deposition (CVD) and MOCVD techniques are now able to provide these materials at wafer-scale [88,89], challenges remain. The primary challenges are ensuring consistent thickness over large areas and the successful transfer of these layers from the growth substrate on to suitable substrates for further fabrication (e.g. lithography). For example, the growth of graphene by CVD can produce large areas of mostly single layer graphene on copper substrates, and while this is impressive, most applications require single layer control over the graphene thickness and in many cases control over the orientation. Mapping approaches have been devised to assess the properties of the transferred film [162], but clearly more uniform growth is the preferred solution.

Looking to the future, the range of new topological semimetals which have been proposed or recently discovered offer yet more potential for terahertz technology. However, there is still demand for better technology in this part of the spectrum, most notably in the sources. While QCLs have markedly improved the range of laser sources available, they are still hampered by lower operating temperatures and a corresponding pulsed operation, despite the lack of a fundamental limit on the operating temperature. Modern compact Sterling and Peltier coolers will enable the use of terahertz QCLs in many applications, but for wider adoption, new materials may be required for laser operation at terahertz frequencies.

## Data Availability

This article has no additional data.

## References

[1] Adams AR, O’Reilly EP, Silver M. 1999 Chapter 2 -Strained layer quantum well lasers. In Semiconductor lasers I (ed. E Kapon), pp. 123–176. San Diego, CA: Academic Press. (10.1016/B978-012397630-7/50002-0)

[2] Lee YS. 2009 Principles of terahertz science and technology. New York, NY: Springer. (10.1007/978-0-387-09540-0_5)

[3] Rubens H, Nichols EF. 1897 Heat rays of great wave length. Phys. Rev. **4**, 314–323. (10.1103/PhysRevSeriesI.4.314)

[4] de Arrieta IG. 2022 Beyond the infrared: a centenary of Heinrich Rubens’s death. Eur. Phys. J. H **47**, 11. (10.1140/epjh/s13129-022-00044-x)

[5] Auston DH, Cheung KP, Smith PR. 1984 Picosecond photoconducting Hertzian dipoles. Appl. Phys. Lett. **45**, 284–286. (10.1063/1.95174)

[6] Gregory IS, Baker C, Tribe WR, Evans MJ, Beere HE, Linfield EH, Davies AG, Missous M. 2003 High resistivity annealed low-temperature GaAs with 100 fs lifetimes. Appl. Phys. Lett. **83**, 4199–4201. (10.1063/1.1628389)

[7] Gupta S, Whitaker JF, Mourou GA. 1992 Ultrafast carrier dynamics in III-V semiconductors grown by molecular-beam epitaxy at very low substrate temperatures. IEEE J. Quantum Electron. **28**, 2464–2472. (10.1109/3.159553)

[8] Davies AG, Burnett AD, Fan W, Linfield EH, Cunningham JE. 2008 Terahertz spectroscopy of explosives and drugs. Mater. Today **11**, 18–26. (10.1016/s1369-7021(08)70016-6)

[9] Davies AG. 2000 Quantum electronics: the physics and technology of low-dimensional electronic systems into the new millennium. Phil. Trans. R. Soc. A **358**, 151–172. (10.1098/rsta.2000.0525)

[10] Faist J, Capasso F, Sivco DL, Sirtori C, Hutchinson AL, Cho AY. 1994 Quantum cascade laser. Science **264**, 553–556. (10.1126/science.264.5158.553) 17732739

[11] Köhler R, Tredicucci A, Beltram F, Beere HE, Linfield EH, Davies AG, Ritchie DA, Iotti RC, Rossi F. 2002 Terahertz semiconductor-heterostructure laser. Nature **417**, 156–159. (10.1038/417156a) 12000955

[12] Klitzing K v., Dorda G, Pepper M. 1980 New method for high-accuracy determination of the fine-structure constant based on quantized Hall resistance. Phys. Rev. Lett. **45**, 494–497. (10.1103/physrevlett.45.494)

[13] Tsui DC, Stormer HL, Gossard AC. 1982 Two-dimensional magnetotransport in the extreme quantum limit. Phys. Rev. Lett. **48**, 1559–1562. (10.1103/physrevlett.48.1559)

[14] Berggren KF, Pepper M. 2010 Electrons in one dimension. Phil. Trans. R. Soc. A **368**, 1141–1162. (10.1098/rsta.2009.0226) 20123751 PMC3263805

[15] Pohl UW. 2020 Epitaxy of Semiconductors. In Graduate texts in physics. London, UK: Springer Nature. (10.1007/978-3-030-43869-2)

[16] Li LH, Chen L, Freeman JR, Salih M, Dean P, Davies AG, Linfield EH. 2017 Multi‐watt high‐power THz frequency quantum cascade lasers. Electron. Lett. **53**, 799–800. (10.1049/el.2017.0662)

[17] Beere HE *et al*. 2005 MBE growth of terahertz quantum cascade lasers. J. Cryst. Growth **278**, 756–764. (10.1016/j.jcrysgro.2004.12.172)

[18] Beere HE, Freeman JR, Marshall OP, Worrall CH, Ritchie DA. 2009 The reproducibility and transferability of a THz quantum cascade laser design between two MBE growth manufacturers’ platforms. J. Cryst. Growth **311**, 1923–1928. (10.1016/j.jcrysgro.2008.11.053)

[19] Scalari G, Ajili L, Faist J, Beere H, Linfield E, Ritchie D, Davies G. 2003 Far-infrared (λ≃87 μm) bound-to-continuum quantum-cascade lasers operating up to 90 K. Appl. Phys. Lett. **82**, 3165–3167. (10.1063/1.1571653)

[20] Williams BS, Kumar S, Callebaut H, Hu Q, Reno JL. 2003 Terahertz quantum-cascade laser operating up to 137 K. Appl. Phys. Lett. **83**, 5142–5144. (10.1063/1.1635657)

[21] Khalatpour A, Tam MC, Addamane SJ, Reno J, Wasilewski Z, Hu Q. 2023 Enhanced operating temperature in terahertz quantum cascade lasers based on direct phonon depopulation. Appl. Phys. Lett **122**, 161101. (10.1063/5.0144705)

[22] Amanti MI, Scalari G, Terazzi R, Fischer M, Beck M, Faist J, Rudra A, Gallo P, Kapon E. 2009 Bound-to-continuum terahertz quantum cascade laser with a single-quantum-well phonon extraction/injection stage. New J. Phys. **11**, 125022. (10.1088/1367-2630/11/12/125022)

[23] Wienold M, Röben B, Schrottke L, Sharma R, Tahraoui A, Biermann K, Grahn HT. 2014 High-temperature, continuous-wave operation of terahertz quantum-cascade lasers with metal-metal waveguides and third-order distributed feedback. Opt. Express **22**, 3334–3348. (10.1364/OE.22.003334) 24663624

[24] Wen B, Ban D. 2021 High-temperature terahertz quantum cascade lasers. Prog. Quantum Electron. **80**, 100363. (10.1016/j.pquantelec.2021.100363)

[25] Miyoshi T, Wang KX (Cathy), Lin TT. 2023 Doping study of two-well resonant-phonon terahertz quantum cascade lasers part I: doping profile dependence. Jpn. J. Appl. Phys. (2008). **62**, 032002. (10.35848/1347-4065/acbdd8)

[26] Fischer M, Scalari G, Walther Ch, Faist J. 2009 Terahertz quantum cascade lasers based on In0.53Ga0.47As/In0.52Al0.48As/InP. J. Cryst. Growth **311**, 1939–1943. (10.1016/j.jcrysgro.2008.10.090)

[27] Deutsch C *et al*. 2013 InGaAs/GaAsSb/InP terahertz quantum cascade lasers. J. Infrared Millim. Terahertz Waves **34**, 374–385. (10.1007/s10762-013-9991-5)

[28] Ye F *et al*. 2023 M-plane GaN terahertz quantum cascade laser structure design and doping effect for resonant-phonon and phonon-scattering-injection schemes. Sci. Rep. **13**, 11394. (10.1038/s41598-023-38627-3) 37452198 PMC10349038

[29] Atić A, Wang X, Vuković N, Stanojević N, Demić A, Indjin D, Radovanović J. 2024 Resonant tunnelling and intersubband optical properties of ZnO/ZnMgO semiconductor heterostructures: impact of doping and layer structure variation. Materials **17**, 927. (10.3390/ma17040927) 38399178 PMC10890650

[30] Cimbri D, Wang J, Al-Khalidi A, Wasige E. 2022 Resonant tunneling diodes high-speed terahertz wireless communications - a review. IEEE Trans. Terahertz Sci. Technol. **12**, 226–244. (10.1109/tthz.2022.3142965)

[31] Choi W, O KK. 2024 Enabling applications of electromagnetic waves at 0.3–1.0 THz using silicon electronic integrated circuits. ACS Photonics **11**, 1362–1375. (10.1021/acsphotonics.3c01129) 38645999 PMC11027913

[32] Gao L, Chan CH. 2022 A 0.68–0.72-THz 2-D scalable radiator array with –3-dBm radiated power and 27.3-dBm EIRP in 65-nm CMOS. IEEE J. Solid State Circuits **57**, 3114–3124. (10.1109/jssc.2022.3178721)

[33] Choi KK. 1997 The physics of quantum well infrared photodetectors. vol. 7. Singapore: World Scientific.

[34] Schneider H, Liu HC. 2007 Quantum well infrared photodetectors. Springer series in optical sciences. Heidelberg, Germany: Springer.

[35] Liu HC, Song CY, SpringThorpe AJ, Cao JC. 2004 Terahertz quantum-well photodetector. Appl. Phys. Lett. **84**, 4068–4070. (10.1063/1.1751620)

[36] Liu X, Liao K, Zhou X, Li Z, Li N. 2013 A 46μm AlGsAs/GaAs terahertz quantum-well infrared photodetector. In ISPDI 2013 - Fifth Int. Symposium on Photoelectronic Detection and Imaging, Beijing, China. vol. 8909. SPIE. (10.1117/12.2034762)

[37] Scalari G, Graf M, Hofstetter D, Faist J, Beere H, Ritchie D. 2006 A THz quantum cascade detector in a strong perpendicular magnetic field. Semicond. Sci. Technol. **21**, 1743–1746. (10.1088/0268-1242/21/12/042)

[38] Yang H, Zheng Y, Tang Z, Li N, Zhou X, Chen P, Wang J. 2020 MBE growth of high performance very long wavelength InGaAs/GaAs quantum well infrared photodetectors. J. Phys. D **53**, 135110. (10.1088/1361-6463/ab66d7)

[39] Quach P *et al*. 2022 A 5.7 THz GaN/AlGaN quantum cascade detector based on polar step quantum wells. Appl. Phys. Lett. **120**. (10.1063/5.0086641)

[40] Zibik EA *et al*. 2009 Long lifetimes of quantum-dot intersublevel transitions in the terahertz range. Nat. Mater. **8**, 803–807. (10.1038/nmat2511) 19684587

[41] Huang G, Yang J, Bhattacharya P, Ariyawansa G, Perera AGU. 2008 A multicolor quantum dot intersublevel detector with photoresponse in the terahertz range. Appl. Phys. Lett **92**, 171103. (10.1063/1.2830994)

[42] Yachmenev AE, Khabibullin RA, Ponomarev DS. 2022 Recent advances in THz detectors based on semiconductor structures with quantum confinement: a review. J. Phys. D **55**, 193001. (10.1088/1361-6463/ac43dd)

[43] Bai P *et al*. 2022 Broadband and photovoltaic THz/IR response in the GaAs-based ratchet photodetector. Sci. Adv. **8**, eabn2031. (10.1126/sciadv.abn2031) 35613269 PMC9132437

[44] Li H *et al*. 2017 6.2-GHz modulated terahertz light detection using fast terahertz quantum well photodetectors. Sci. Rep **7**, 3452. (10.1038/s41598-017-03787-6) 28615654 PMC5471194

[45] Dely H *et al*. 2023 High bitrate data transmission in the 8-14 µm atmospheric window using an external Stark-effect modulator with digital equalization. Opt. Express **31**, 7259–7264. (10.1364/oe.474209) 36859861

[46] Büchel D *et al*. 2015 4.7-THz superconducting hot electron bolometer waveguide mixer. IEEE Trans. THz Sci. Technol **5**, 207–214. (10.1109/TTHZ.2014.2382434)

[47] Yadav R, Ludwig F, Faridi FR, Klopf JM, Roskos HG, Preu S, Penirschke A. 2023 State-of-the-art room temperature operable zero-bias Schottky diode-based terahertz detector up to 5.56 THz. Sensors **23**, 3469. (10.3390/s23073469) 37050531 PMC10098974

[48] Kou W, Liang S, Zhou H, Dong Y, Gong S, Yang Z, Zeng H. 2022 A review of terahertz sources based on planar Schottky diodes. Chinese J. Electron. **31**, 467–487. (10.1049/cje.2021.00.302)

[49] 2024 CST GmbH. See https://acst.de/.

[50] 2024 Virginia Diodes Inc. See https://www.vadiodes.com/.

[51] Palaferri D, Todorov Y, Chen YN, Madeo J, Vasanelli A, Li LH, Davies AG, Linfield EH, Sirtori C. 2015 Patch antenna terahertz photodetectors. Appl. Phys. Lett **106**, 161102. (10.1063/1.4918983)

[52] Jeannin M *et al*. 2020 High temperature metamaterial terahertz quantum detector. Appl. Phys. Lett **117**, 251102. (10.1063/5.0033367)

[53] Rodriguez E *et al*. 2022 Metamaterial engineering for optimized photon absorption in unipolar quantum devices. Opt. Express **30**, 20515–20531. (10.1364/OE.456318) 36224794

[54] Degl’Innocenti R, Lin H, Navarro-Cía M. 2022 Recent progress in terahertz metamaterial modulators. Nanophotonics **11**, 1485–1514. (10.1515/nanoph-2021-0803) 39635280 PMC11501865

[55] Lewis RA. 2019 A review of terahertz detectors. J. Phys. D **52**, 433001. (10.1088/1361-6463/ab31d5)

[56] Dyakonov M, Shur M. 1996 Detection, mixing, and frequency multiplication of terahertz radiation by two-dimensional electronic fluid. IEEE Trans. Electron Devices **43**, 380–387. (10.1109/16.485650)

[57] Knap W, Deng Y, Rumyantsev S, Shur MS. 2002 Resonant detection of subterahertz and terahertz radiation by plasma waves in submicron field-effect transistors. Appl. Phys. Lett. **81**, 4637–4639. (10.1063/1.1525851)

[58] Knap W *et al*. 2002 Nonresonant detection of terahertz radiation in field effect transistors. J. Appl. Phys. **91**, 9346–9353. (10.1063/1.1468257)

[59] Javadi E *et al*. 2019 Terahertz detection with a low-cost packaged GaAs high-electron-mobility transistor. IEEE Trans. THz. Sci. Technol **9**, 27–37. (10.1109/TTHZ.2018.2877908)

[60] Regensburger S *et al*. 2018 Broadband terahertz detection with zero-bias field-effect transistors between 100 GHz and 11.8 THz with a noise equivalent power of 250 pW/√Hz at 0.6 THz. IEEE Trans. THz. Sci. Technol **8**, 465–471. (10.1109/TTHZ.2018.2843535)

[61] Lü L. 2015 Mapping an on-chip terahertz antenna by a scanning near-field probe and a fixed field-effect transistor. Chin. Phys. B **24**, 028504. (10.1088/1674-1056/24/2/028504)

[62] Sun JD *et al*. 2015 The effect of symmetry on resonant and nonresonant photoresponses in a field-effect terahertz detector. Appl. Phys Lett **106**, 031119. (10.1063/1.4906536)

[63] Sun JD, Qin H, Lewis RA, Sun YF, Zhang XY, Cai Y, Wu DM, Zhang BS. 2012 Probing and modelling the localized self-mixing in a GaN/AlGaN field-effect terahertz detector. Appl. Phys. Lett. **100**, 173513. (10.1063/1.4705306)

[64] Boppel S. 2016 0.25-μm GaN TeraFETs optimized as THz power detectors and intensity-gradient sensors. IEEE Trans. THz. Sci. Technol **6**, 348–350. (10.1109/TTHZ.2016.2520202)

[65] Hou H, Liu Z, Teng J, Palacios T, Chua SJ. 2017 A sub-terahertz broadband detector based on a GaN high-electron-mobility transistor with nanoantennas. Appl. Phys. Express **10**, 014101. (10.7567/APEX.10.014101) PMC5399372 28429745

[66] Liu Z *et al*. 2017 A CMOS fully integrated 860-GHz terahertz sensor. IEEE Trans THz Sci Technol **7**, 455–465. (10.1109/TTHZ.2017.2692040)

[67] Ali M, Perenzoni M. 2018 A readout channel optimized for minimum NEP of a FET-based THz pixel. J. Infrared Millim. Terahertz Waves **39**, 1221–1235. (10.1007/s10762-018-0531-1)

[68] Zdanevičius J. 2018 Field-effect transistor based detectors for power monitoring of THz quantum cascade lasers. IEEE Trans THz Sci Technol **8**, 613–621. (10.1109/TTHZ.2018.2871360)

[69] Huang TN *et al*. 2016 A 65 nm CMOS LNA for bolometer application. J Infrared Millim. THz Waves **37**, 356–372. (10.1007/s10762-016-0244-2)

[70] Varlamava V *et al*. 2016 CMOS-compatible room-temperature rectifier toward terahertz radiation detection. J. Infrared Millim. THz Waves **37**, 737–752. (10.1007/s10762-016-0265-x)

[71] Boppel S *et al*. 2012 CMOS integrated antenna-coupled field-effect transistors for the detection of radiation from 0.2 to 4.3 THz. IEEE Trans. Microw. Theory Tech. **60**, 3834–3843. (10.1109/tmtt.2012.2221732)

[72] Čibiraite D, Wan M, Lisauskas A, Ramer A, Chevtchenko S, Heinrich W, Roskos HG, Sheridan JT, Krozer V. 2019 TeraFET multi-pixel THz array for a confocal imaging system. In 2019 44th Int. Conf. on Infrared, Millimeter, and Terahertz Waves (IRMMW-THz), Paris, France. IEEE. (10.1109/IRMMW-THz.2019.8874217)

[73] Holstein J *et al*. 2024 8x8 patch-antenna-coupled TeraFET detector array for terahertz quantum-cascade-laser applications. IEEE Trans. Terahertz Sci. Technol. **14**, 799–807. (10.1109/TTHZ.2024.3438429)

[74] Javadi E, But DB, Ikamas K, Zdanevičius J, Knap W, Lisauskas A. 2021 Sensitivity of field-effect transistor-based terahertz detectors. Sensors **21**, 2909. (10.3390/s21092909) 33919219 PMC8122696

[75] Regensburger S, Winnerl S, Klopf JM, Lu H, Gossard AC, Preu S. 2019 Picosecond-scale terahertz pulse characterization with field-effect transistors. IEEE Trans. Terahertz Sci. Technol. **9**, 262–271. (10.1109/tthz.2019.2903630)

[76] Wood CD, Hatem O, Cunningham JE, Linfield EH, Davies AG, Cannard PJ, Robertson MJ, Moodie DG. 2010 Terahertz emission from metal-organic chemical vapor deposition grown Fe:InGaAs using 830 nm to 1.55 μm excitation. Appl. Phys. Lett **96**, 194104. (10.1063/1.3427191)

[77] Dietz RJB, Globisch B, Roehle H, Stanze D, Göbel T, Schell M. 2014 Influence and adjustment of carrier lifetimes in InGaAs/InAlAs photoconductive pulsed terahertz detectors: 6 THz bandwidth and 90dB dynamic range. Opt. Express **22**, 19411–19422. (10.1364/oe.22.019411) 25321025

[78] Bacon DR, Madéo J, Dani KM. 2021 Photoconductive emitters for pulsed terahertz generation. J. Opt. **23**, 064001. (10.1088/2040-8986/abf6ba)

[79] Papaioannou ET, Beigang R. 2021 THz spintronic emitters: a review on achievements and future challenges. Nanophotonics **10**, 1243–1257. (10.1515/nanoph-2020-0563)

[80] Beaurepaire E, Turner GM, Harrel SM, Beard MC, Bigot JY, Schmuttenmaer CA. 2004 Coherent terahertz emission from ferromagnetic films excited by femtosecond laser pulses. Appl. Phys. Lett. **84**, 3465–3467. (10.1063/1.1737467)

[81] Kampfrath T *et al*. 2013 Terahertz spin current pulses controlled by magnetic heterostructures. Nat. Nanotechnol. **8**, 256–260. (10.1038/nnano.2013.43) 23542903

[82] Wang M, Zhang Y, Guo L, Lv M, Wang P, Wang X. 2022 Spintronics based terahertz sources. Crystals **12**, 1661. (10.3390/cryst12111661)

[83] Seifert T *et al*. 2016 Efficient metallic spintronic emitters of ultrabroadband terahertz radiation. Nat. Photonics **10**, 483–488. (10.1038/nphoton.2016.91)

[84] Sasaki Y *et al*. 2019 Effect of Co and Fe stoichiometry on terahertz emission from Ta/CoFeB/MgO thin films. Phys. Rev. B **100**, 140406. (10.1103/PhysRevB.100.140406)

[85] Rouzegar R *et al*. 2023 Broadband spintronic terahertz source with peak electric fields exceeding 1.5 MV/cm. Phys. Rev. Appl. **19**, 034018. (10.1103/physrevapplied.19.034018)

[86] Novoselov KS, Geim AK, Morozov SV, Jiang D, Zhang Y, Dubonos SV, Grigorieva IV, Firsov AA. 2004 Electric field effect in atomically thin carbon films. Science **306**, 666–669. (10.1126/science.1102896) 15499015

[87] Novoselov KS *et al*. 2007 Room-temperature quantum Hall effect in graphene. Science **315**, 1379. (10.1126/science.1137201) 17303717

[88] Lei Y *et al*. 2022 Graphene and beyond: recent advances in two-dimensional materials synthesis, properties, and devices. ACS Nanosci. Au **2**, 450–485. (10.1021/acsnanoscienceau.2c00017) 36573124 PMC9782807

[89] Shanmugam V *et al*. 2022 A review of the synthesis, properties, and applications of 2D materials. Part. Part. Syst. Charact. **39**, 2200031. (10.1002/ppsc.202200031)

[90] Avouris P, Heinz TF, Low T (eds). 2017 2D materials properties and devices. In 2D materials: properties and devices pp. i–i. Cambridge, UK: Cambridge University Press. (10.1017/9781316681619)

[91] Castro Neto AH, Guinea F, Peres NMR, Novoselov KS, Geim AK. 2009 The electronic properties of graphene. Rev. Mod. Phys. **81**, 109–162. (10.1103/RevModPhys.81.109)

[92] Falkovsky LA. 2008 Optical properties of graphene. J. Phys. **129**, 012004. (10.1088/1742-6596/129/1/012004)

[93] Rogalski A. 2019 Graphene-based materials in the infrared and terahertz detector families: a tutorial. Adv. Opt. Photonics **11**, 314–379. (10.1364/AOP.11.000314)

[94] Viti L, Vitiello MS. 2021 Tailored nano-electronics and photonics with two-dimensional materials at terahertz frequencies. J. Appl. Phys **130**, 170903. (10.1063/5.0065595)

[95] Lee GH *et al*. 2020 Graphene-based Josephson junction microwave bolometer. Nature **586**, 42–46. (10.1038/s41586-020-2752-4) 32999482

[96] Kokkoniemi R *et al*. 2020 Bolometer operating at the threshold for circuit quantum electrodynamics. Nature **586**, 47–51. (10.1038/s41586-020-2753-3) 32999484

[97] Gunyhó AM *et al*. 2024 Single-shot readout of a superconducting qubit using a thermal detector. Nat. Electron. **7**, 288–298. (10.1038/s41928-024-01147-7) PMC13545009 42701336

[98] Duan J, Wang X, Lai X, Li G, Watanabe K, Taniguchi T, Zebarjadi M, Andrei EY. 2016 High thermoelectricpower factor in graphene/hBN devices. Proc. Natl Acad. Sci. USA **113**, 14272–14276. (10.1073/pnas.1615913113) 27911824 PMC5167211

[99] Viti L *et al*. 2021 Thermoelectric graphene photodetectors with sub-nanosecond response times at terahertz frequencies. Nanophotonics **10**, 89–98. (10.1515/nanoph-2020-0255)

[100] Qin H, Sun J, Liang S, Li X, Yang X, He Z, Yu C, Feng Z. 2017 Room-temperature, low-impedance and high-sensitivity terahertz direct detector based on bilayer graphene field-effect transistor. Carbon N Y **116**, 760–765. (10.1016/j.carbon.2017.02.037)

[101] Vicarelli L, Vitiello MS, Coquillat D, Lombardo A, Ferrari AC, Knap W, Polini M, Pellegrini V, Tredicucci A. 2012 Graphene field-effect transistors as room-temperature terahertz detectors. Nat. Mater. **11**, 865–871. (10.1038/nmat3417) 22961203

[102] Vizbaras D, Ikamas K, Pralgauskaitė S, Matukas J, Generalov AA, Lisauskas A. 2022 Optimization of terahertz detectors based on graphene field effect transistors by high impedance antennae. Lith. J. Phys **62**, 254–266. (10.3952/physics.v62i4.4822)

[103] Liu C, Wang L, Chen X, Politano A, Wei D, Chen G, Tang W, Lu W, Tredicucci A. 2018 Room‐temperature high‐gain long‐wavelength photodetector via optical–electrical controlling of hot carriers in graphene. Adv. Opt. Mater. **6**, 1800836. (10.1002/adom.201800836)

[104] Ludwig F, Generalov A, Holstein J, Murros A, Viisanen K, Prunnila M, Roskos HG. 2024 Terahertz detection with graphene FETs: photothermoelectric and resistive self-mixing contributions to the detector response. ACS Appl. Electron. Mater. **6**, 2197–2212. (10.1021/acsaelm.3c01511)

[105] Malevich Y, Ergoktas MS, Bakan G, Steiner P, Kocabas C. 2020 Video-speed graphene modulator arrays for terahertz imaging applications. ACS Photonics **7**, 2374–2380. (10.1021/acsphotonics.0c00991)

[106] Zaman AM *et al*. 2022 Terahertz metamaterial optoelectronic modulators with GHz reconfiguration speed. IEEE Trans. Terahertz Sci. Technol. **12**, 520–526. (10.1109/tthz.2022.3178875)

[107] Peng X, Yan Y. 2021 Graphene saturable absorbers applications in fiber lasers. J. Eur. Opt. Soc. Rapid Publ. **17**, 16. (10.1186/s41476-021-00163-w)

[108] Bianchi V *et al*. 2017 Terahertz saturable absorbers from liquid phase exfoliation of graphite. Nat. Commun. **8**, 15763. (10.1038/ncomms15763) 28643788 PMC5481741

[109] Hafez HA *et al*. 2018 Extremely efficient terahertz high-harmonic generation in graphene by hot Dirac fermions. Nature **561**, 507–511. (10.1038/s41586-018-0508-1) 30202091

[110] Hafez HA, Kovalev S, Tielrooij K, Bonn M, Gensch M, Turchinovich D. 2020 Terahertz nonlinear optics of graphene: from saturable absorption to high‐harmonics generation. Adv. Opt. Mater **8**, 1900771. (10.1002/adom.201900771)

[111] Di Gaspare A *et al*. 2024 Compact terahertz harmonic generation in the Reststrahlenband using a graphene-embedded metallic split ring resonator array. Nat. Commun. **15**, 2312. (10.1038/s41467-024-45267-2) 38485950 PMC10940712

[112] Manzeli S, Ovchinnikov D, Pasquier D, Yazyev OV, Kis A. 2017 2D transition metal dichalcogenides. Nat. Rev. Mater. **2**, 17033. (10.1038/natrevmats.2017.33)

[113] Villaos RAB, Crisostomo CP, Huang ZQ, Huang SM, Padama AAB, Albao MA, Lin H, Chuang FC. 2019 Thickness dependent electronic properties of Pt dichalcogenides. Npj 2D Mater. Appl. **3**, 2. (10.1038/s41699-018-0085-z)

[114] Ciarrocchi A, Avsar A, Ovchinnikov D, Kis A. 2018 Thickness-modulated metal-to-semiconductor transformation in a transition metal dichalcogenide. Nat. Commun. **9**, 919. (10.1038/s41467-018-03436-0) 29500434 PMC5834615

[115] Jakhar A, Kumar P, Moudgil A, Dhyani V, Das S. 2020 Optically pumped broadband terahertz modulator based on nanostructured PtSe_2_ thin films. Adv. Opt. Mater. **8**, 1901714. (10.1002/adom.201901714)

[116] Xu H *et al*. 2019 PtTe2‐based type‐II Dirac semimetal and its van der Waals heterostructure for sensitive room temperature terahertz photodetection. Small **15**, 1903362. (10.1002/smll.201903362) 31736239

[117] Dong Z *et al*. 2021 Highly efficient, ultrabroad PdSe_2_ phototransistors from visible to terahertz driven by mutiphysical mechanism. ACS Nano **15**, 20403–20413. (10.1021/acsnano.1c08756) 34780146

[118] Shein K *et al*. 2024 Fundamental limits of few-layer NbSe2 microbolometers at terahertz frequencies. Nano. Lett. **24**, 2282–2288. (10.1021/acs.nanolett.3c04493) 38345381

[119] Li J, Ma W, Jiang L, Yao N, Deng J, Qiu Q, Shi Y, Zhou W, Huang Z. 2022 High performance of room-temperature NbSe_2_ terahertz photoelectric detector. ACS Appl. Mater. Interfaces **14**, 14331–14341. (10.1021/acsami.2c00175) 35289598

[120] Chen S, Fan F, Miao Y, He X, Zhang K, Chang S. 2016 Ultrasensitive terahertz modulation by silicon-grown MoS_2_ nanosheets. Nanoscale **8**, 4713–4719. (10.1039/C5NR08101G) 26856303

[121] Yadav P, Xinhou C, Bhatt S, Das S, Yang H, Mishra R. 2024 Highly efficient spintronic terahertz emitter utilizing a large spin Hall conductivity of type-II Dirac semimetal PtTe2. Nano Lett **24**, 2376–2383. (10.1021/acs.nanolett.3c04986) 38329912

[122] Antonatos N, Šturala J, Mazánek V, Sedmidubský D, Veselý M, Růžička K, Hejtmánek J, Levinsky P, Sofer Z. 2022 Black phosphorus: fundamental properties and influence of impurities induced by its synthesis. ACS Appl. Mater. Interfaces **14**, 34867–34874. (10.1021/acsami.2c08714) 35856643

[123] Zhang Y, Wang J, Liu Q, Gu S, Sun Z, Chu PK, Yu X. 2020 The electrical, thermal, and thermoelectric properties of black phosphorus. APL Mater **8**, 120903. (10.1063/5.0027244)

[124] Wang L *et al*. 2017 Toward sensitive room‐temperature broadband detection from infrared to terahertz with antenna‐integrated black phosphorus photoconductor. Adv. Funct. Mater **27**, 1604414. (10.1002/adfm.201604414)

[125] Viti L, Hu J, Coquillat D, Politano A, Knap W, Vitiello MS. 2016 Efficient terahertz detection in black-phosphorus nano-transistors with selective and controllable plasma-wave, bolometric and thermoelectric response. Sci. Rep. **6**, 20474. (10.1038/srep20474) 26847823 PMC4742799

[126] Pogna EAA, Pistore V, Viti L, Li L, Davies AG, Linfield EH, Vitiello MS. 2024 Near-field detection of gate-tunable anisotropic plasmon polaritons in black phosphorus at terahertz frequencies. Nat. Commun. **15**, 2373. (10.1038/s41467-024-45264-5) 38490988 PMC10943022

[127] Wu Z, Lyu Y, Zhang Y, Ding R, Zheng B, Yang Z, Lau SP, Chen XH, Hao J. 2021 Large-scale growth of few-layer two-dimensional black phosphorus. Nat. Mater. **20**, 1203–1209. (10.1038/s41563-021-01001-7) 33972761

[128] Jia L, Wu J, Zhang Y, Qu Y, Jia B, Chen Z, Moss DJ. 2022 Fabrication technologies for the on‐chip integration of 2D materials. Small Methods **6**, 2101435. (10.1002/smtd.202101435) 34994111

[129] Thouless DJ, Kohmoto M, Nightingale MP, den Nijs M. 1982 Quantized Hall conductance in a two-dimensional periodic potential. Phys. Rev. Lett. **49**, 405. (10.1103/physrevlett.49.405)

[130] Kohmoto M. 1985 Topological invariant and the quantization of the Hall conductance. Ann. Phys. **160**, 343–354. (10.1016/0003-4916(85)90148-4)

[131] Kane CL, Mele EJ. 2005 Z2 topological order and the quantum spin Hall effect. Phys. Rev. Lett. **95**, 146802. (10.1103/PhysRevLett.95.146802) 16241681

[132] Kane CL, Mele EJ. 2005 Quantum spin Hall effect in graphene. Phys. Rev. Lett. **95**, 226801. (10.1103/physrevlett.95.226801) 16384250

[133] Tokura Y, Yasuda K, Tsukazaki A. 2019 Magnetic topological insulators. Nat. Rev. Phys. **1**, 126–143. (10.1038/s42254-018-0011-5)

[134] Bianchi M, Guan D, Bao S, Mi J, Iversen BB, King PDC, Hofmann P. 2010 Coexistence of the topological state and a two-dimensional electron gas on the surface of Bi2Se3. Nat. Commun. **1**, 128. (10.1038/ncomms1131) 21119641

[135] Kozlov DA, Kvon ZD, Olshanetsky EB, Mikhailov NN, Dvoretsky SA, Weiss D. 2014 Transport properties of a 3D topological insulator based on a strained high-mobility HgTe film. Phys. Rev. Lett. **112**, 196801. (10.1103/physrevlett.112.196801) 24877958

[136] Valdés Aguilar R *et al*. 2012 Terahertz response and colossal Kerr rotation from the surface states of the topological insulator Bi2Se3. Phys. Rev. Lett. **108**, 087403. (10.1103/PhysRevLett.108.087403) 22463570

[137] McIver JW *et al*. 2012 Theoretical and experimental study of second harmonic generation from the surface of the topological insulator Bi2Se3. Phys. Rev. B **86**, 035327. (10.1103/PhysRevB.86.035327)

[138] Dziom V *et al*. 2017 Observation of the universal magnetoelectric effect in a 3D topological insulator. Nat. Commun. **8**, 15197. (10.1038/ncomms15197) 28504268 PMC5440727

[139] Knox CS *et al*. 2022 Effects of structural ordering on infrared active vibrations within Bi2(Te(1-x)Se(x))3. Phys. Rev. B **106**, 245203. (10.1103/PhysRevB.106.245203)

[140] Olbrich P *et al*. 2014 Room-temperature high-frequency transport of Dirac fermions in epitaxially grown Sb2Te3 - and Bi2Te3 -based topological insulators. Phys. Rev. Lett. **113**, 096601. (10.1103/PhysRevLett.113.096601) 25215999

[141] Hamh SY. 2016 Helicity-dependent photocurrent in a Bi2Se3 thin film probed by terahertz emission spectroscopy. Phys. Rev. B **94**, 161405. (10.1103/PhysRevB.94.161405)

[142] Zhu LG, Kubera B, Fai Mak K, Shan J. 2015 Effect of surface states on terahertz emission from the Bi2Se3 surface. Sci. Rep **5**, 10308. (10.1038/srep10308) 25988722 PMC4437309

[143] Tu CM *et al*. 2017 Helicity-dependent terahertz emission spectroscopy of topological insulator Sb2Te3 thin films. Phys. Rev. B **96**, 195407. (10.1103/PhysRevB.96.195407)

[144] Onishi Y. 2015 Ultrafast carrier relaxation through Auger recombination in the topological insulator Bi1.5Sb0.5Te1.7Se1.3. Phys. Rev. B **91**, 085306. (10.1103/PhysRevB.91.085306)

[145] Fang Z *et al*. 2019 Nonlinear terahertz emission in the three-dimensional topological insulator Bi2Te3 by terahertz emission spectroscopy. Appl. Phys. Lett **115**, 191102. (10.1063/1.5097335)

[146] Tielrooij KJ *et al*. 2022 Milliwatt terahertz harmonic generation from topological insulator metamaterials. Light **11**, 315. (10.1038/s41377-022-01008-y) PMC9622918 36316317

[147] Sinha A, Mithun KP, Sood AK. 2023 Time-resolved second-harmonic generation in topological insulator Bi2Te3 competing contributions from Dirac surface states, surface photovoltage, and band bending. ACS Photonics **10**, 3944–3954. (10.1021/acsphotonics.3c00722)

[148] Giorgianni F *et al*. 2016 Strong nonlinear terahertz response induced by Dirac surface states in Bi2Se3 topological insulator. Nat. Commun **7**, 11421. (10.1038/ncomms11421) 27113395 PMC4853424

[149] Kovalev S *et al*. 2021 Terahertz signatures of ultrafast Dirac fermion relaxation at the surface of topological insulators. Npj Quantum Mater **6**, 84. (10.1038/s41535-021-00384-9)

[150] He M, Sun H, He QL. 2019 Topological insulator: spintronics and quantum computations. Front. Phys **14**, 43401. (10.1007/s11467-019-0893-4)

[151] Kuznetsov KA, Tarasenko SA, Kovaleva PM, Kuznetsov PI, Lavrukhin DV, Goncharov YG, Ezhov AA, Ponomarev DS, Kitaeva GKh. 2022 Topological insulator films for terahertz photonics. Nanomaterials **12**, 3779. (10.3390/nano12213779) 36364555 PMC9658460

[152] Park H *et al*. 2021 Enhanced spin-to-charge conversion efficiency in ultrathin Bi2Se3 observed by spintronic terahertz spectroscopy. ACS Appl. Mater. Interfaces **13**, 23153–23160. (10.1021/acsami.1c03168) 33945256

[153] Armitage NP, Mele EJ, Vishwanath A. 2018 Weyl and Dirac semimetals in three-dimensional solids. Rev. Mod. Phys. **90**, 015001. (10.1103/revmodphys.90.015001)

[154] Lv BQ, Qian T, Ding H. 2021 Experimental perspective on three-dimensional topological semimetals. Rev. Mod. Phys. **93**, 025002. (10.1103/revmodphys.93.025002)

[155] Feng X, Zhu J, Wu W, Yang SA. 2021 Two-dimensional topological semimetals. Chin. Phys. B **30**, 107304. (10.1088/1674-1056/ac1f0c)

[156] Li Y *et al*. 2024 Two-dimensional topological semimetals: an emerging candidate for terahertz detectors and on-chip integration. Mater. Horizons **11**, 2572–2602. (10.1039/d3mh02250a) 38482962

[157] Li S, Yu ZM, Yao Y, Yang SA. 2020 Type-II topological metals. Front. Phys. **15**, 1–12. (10.1007/s11467-020-0963-7)

[158] Yao X *et al*. 2021 Thickness-controlled three-dimensional Dirac semimetal for scalable high-performance terahertz optoelectronics. ACS Photonics **8**, 1689–1697. (10.1021/acsphotonics.1c00127)

[159] Dai Z *et al*. 2021 High mobility 3D Dirac semimetal (Cd3As2) for ultrafast photoactive terahertz photonics. Adv. Funct. Mater **31**, 2011011. (10.1002/adfm.202011011)

[160] He Y *et al*. 2024 Selective growth of type‐II Weyl‐semimetal and Van der Waals stacking for sensitive terahertz photodetection. Adv. Funct. Mater **34**, 2311008. (10.1002/adfm.202311008)

[161] 2024 Teraview Limited. See https://teraview.com/coatings/.

[162] Burton OJ, Winter Z, Watanabe K, Taniguchi T, Beschoten B, Stampfer C, Hofmann S. 2023 Putting high-index Cu on the map for high-yield, dry-transferred CVD graphene. ACS Nano **17**, 1229–1238. (10.1021/acsnano.2c09253) 36594782 PMC9878973

